# Chiral phenoxyacetic acid analogues inhibit colon cancer cell proliferation acting as PPARγ partial agonists

**DOI:** 10.1038/s41598-019-41765-2

**Published:** 2019-04-01

**Authors:** Lina Sabatino, Pamela Ziccardi, Carmen Cerchia, Livio Muccillo, Luca Piemontese, Fulvio Loiodice, Vittorio Colantuoni, Angelo Lupo, Antonio Lavecchia

**Affiliations:** 1 Dipartimento di Scienze e Tecnologie, Università del Sannio, via Port’Arsa 11, 82100 Benevento, Italy; 2 Dipartimento di Farmacia, “Drug Discovery” Laboratory, Università degli Studi di Napoli Federico II, via D. Montesano 49, 80131 Napoli, Italy; 3 Dipartimento Farmacia-Scienze del Farmaco, Università degli Studi di Bari “Aldo Moro”, via Orabona 4, 70125 Bari, Italy

## Abstract

Peroxisome Proliferator-Activated Receptor γ (PPARγ) is an important sensor at the crossroad of diabetes, obesity, immunity and cancer as it regulates adipogenesis, metabolism, inflammation and proliferation. PPARγ exerts its pleiotropic functions upon binding of natural or synthetic ligands. The molecular mechanisms through which PPARγ controls cancer initiation/progression depend on the different mode of binding of distinctive ligands. Here, we analyzed a series of chiral phenoxyacetic acid analogues for their ability to inhibit colorectal cancer (CRC) cells growth by binding PPARγ as partial agonists as assessed in transactivation assays of a *PPARG*-reporter gene. We further investigated compounds (*R*,*S*)**-3**, (*S*)-**3** and (*R*,*S*)**-7** because they combine the best antiproliferative activity and a limited transactivation potential and found that they induce cell cycle arrest mainly *via* upregulation of p21^waf1/cip1^. Interestingly, they also counteract the β-catenin/TCF pathway by repressing c-Myc and cyclin D1, supporting their antiproliferative effect. Docking experiments provided insight into the binding mode of the most active compound (*S*)-**3**, suggesting that its partial agonism could be related to a better stabilization of H3 rather than H11 and H12. In conclusion, we identified a series of PPARγ partial agonists affecting distinct pathways all leading to strong antiproliferative effects. These findings may pave the way for novel therapeutic strategies in CRC.

## Introduction

Colorectal cancer (CRC) is the third most frequent cause of cancer-related death worldwide. In 2017, 95,520 new cases have been estimated in USA with the same incidence in both gender but a higher mortality in men^1^. If diagnosed early, CRC is treatable with a good patient’s prognosis; at later stages, it can spread to other tissues (mostly liver and lung) to form distant metastases and is associated with poor prognosis and high mortality rate^2–4^. The risk to develop CRC and several other cancer types has been correlated with the metabolic syndrome and its associated diseases such as obesity, insulin resistance and type 2 diabetes, another worldwide epidemic condition^5,6^. Thus, significant efforts have been made to identify novel drug targets both for CRC prevention and treatment. The search of new therapeutics represents a great challenge for many investigators and a hopeful chance for millions of patients affected by these diseases.

A number of natural and synthetic compounds function through the binding to specific nuclear receptors (NRs) thus modulating different molecular pathways. NRs constitute a conserved family of ligand-activated transcription factors that regulate the expression of genes involved in a myriad of biological processes such as cell proliferation, metabolism, reproduction and development^7–9^. The peroxisome proliferator-activated receptors (PPARs) are a subgroup of this superfamily, three subtypes of which have been identified so far: PPARα, PPARβ/δ and PPARγ^10^. The three PPAR subtypes regulate the expression of both common and distinct target genes through heterodimerization with members of the retinoid X receptors (RXRs) and binding to the PPAR responsive elements (PPREs) present in the promoter regions^11^. Among the three PPAR subtypes, PPARγ is the most widely studied^12^; from a single gene and two distinct transcriptional start sites, two protein subtypes are synthesized^13^. PPARγ2 is predominantly expressed in adipose tissues^14^, while PPARγ1 is expressed in many tissues of epithelial origin such as intestine, lung, breast, colon, and prostate^15,16^. PPARγ1 upregulation has been detected in malignant tissues such as human prostate and gastric cancer, while PPARγ2 in liposarcoma, suggesting that *PPARG* dysregulation might be involved in cancer pathogenesis^17–19^. Since overexpression is not always related to activation of the downstream pathways, this may be due to either the absence of specific ligands and/or the presence of specific antagonists. In addition, many studies in CRC cell lines and animal models have shown that PPARγ activation inhibits cellular proliferation and angiogenesis, promotes differentiation and apoptosis, leading to postulate a putative role for this receptor as a tumor suppressor gene^20–24^.

A growing list of compounds functions as PPARγ ligands. 15-deoxy-Δ12, 14-prostaglandin J2 (15d-PGJ2), a metabolite of prostaglandin D2, is an endogenous ligand, whereas thiazolidinediones (TZDs) are specific exogenous ligands^25,26^. TZDs have been used for many years in the clinical practice to treat type II diabetes as they reduce blood glucose levels and improve insulin sensitivity. TZDs act as full agonists and have also antitumorigenic activity in a wide variety of cancer cells^27,28^. Both *in vitro* studies and clinical trials of small size, however, have reported controversial results not always tied to beneficial effects^29,30^. Suppression of COX-2 expression with a resulting reduction of PGE_2_ ^31^, matrix metalloproteinase MMP-2 and MMP-9 and increase in their tissue inhibitors TIMP-1 and TIMP-2^31,32^, are some of the beneficial outcomes. Induction of apoptosis associated with halting cell cycle progression and inhibition of genes such as cyclin D1 and c-Myc have also been reported for full agonists^33–35^. Some of the effects exerted by TZDs, in addition, have been related to not-completely elucidated PPARγ -independent mechanisms^36^.

In the present study, we sought to verify whether some chiral phenoxyacetic acid analogues act as PPARγ ligands in a transactivation assay. Indeed, they are part of a longer series of similar compounds previously reported to act as PPARα full agonists^37^; however, some of them exhibited a specific affinity for PPARγ and none for PPARβ/δ. Compounds **1–7** (Table 1) behaved as PPARγ partial agonists in a transactivation assay more reliable than the one previously used. Interestingly, they induce growth inhibition in a PPARγ-dependent manner. Among these compounds, (*R*,*S*)**-3**, (*S*)-**3** and (*R*,*S*)**-7** were further analyzed as they display the better combination of cell growth inhibition and limited transactivation ability. Remarkably, (*R,S*)-**3** and (*S*)-**3** cause cell cycle and proliferation arrest and also inhibit c-Myc and cyclin D1 gene expression thus interfering with the β-catenin/TCF pathway. Docking studies showed that the partial agonism of the most active compound (*S*)-**3** toward PPARγ could be related to an increased stabilization of H3 and lower stabilization of H11 and H12. These results suggest that (*S*)-**3** acts as a PPARγ partial agonist and exerts a strong antiproliferative effects through the combination of a cell cycle arrest, block of a known cell proliferation pathway and induction of apoptosis with beneficial antitumor results.

**Table 1:** Structures of compounds **1**–**7**.


compd	X	R
(*R*)-**1**	(CH_2_)_2_	H
(*R*,*S*)**-2**	(CH_2_)_3_	H
(*R*,*S*)**-3**	(CH_2_)_3_	Cl
(*S*)-**3**	(CH_2_)_3_	Cl
(*R*,*S*)-**4**	(CH_2_)_4_	H
(*R*,*S*)**-5**	(CH_2_)_2_O	Cl
(*R*)-**5**	(CH_2_)_2_O	Cl
(*R*,*S*)**-6**	(CH_2_)_4_O	Cl
(*R*,*S*)**-7**	(CH_2_)_5_O	Cl

## Results

### The chiral phenoxyacetic acid analogues act as PPARγ partial agonists and display antiproliferative capacity

To assess the ability of compounds **1**–**7** (Table 1) to transactivate PPARγ, we transfected a typical PPRE-Luciferase reporter gene in HEK293 cells ectopically expressing a PPARγ1 isoform in addition to the endogenous protein. These cells were selected as a model system because they express low levels of the endogenous PPAR subtypes and a fixed and higher amount of an exogenous, full length PPARγ1; the differences in luciferase activity can, thus, be referred to PPARγ1 and to the different ligands used. Twenty-four hours after transfection, cells were treated for additional 24 hs with increasing doses of each compound (Table 2). All compounds acted as PPARγ partial agonists as they transactivate PPARγ in the range between 40 and 65% with respect to the full agonist rosiglitazone (RGZ) taken as 100%.

**Table 2:** PPARγ transactivation and cell viability activity of chiral phenoxyacetic acid analogues **1**–**7**.

Compounds	EC_50_ (µM)	Efficacy (%)	Proliferation IC_50_ (µM)	Residual viability (%)
(*R*)**-1**	0.64 ± 0.06	45 ± 1.3	19.7 ± 1.7	77 ± 3.1
(*R,S*)**-2**	0.54 ± 0.1	48 ± 2.1	12.1 ± 1.2	70 ± 2.5
(*R,S*)**-3**	0.35 ± 0.04	65 ± 2.4	7.3 ± 0.5	47 ± 2.3
(*S*)-**3**	0.4 ± 0.05	55 ± 1.2	4.8 ± 0.35	31 ± 1.3
(*R,S*)-**4**	0.35 ± 0.07	60 ± 1.1	10.1 ± 0.4	60 ± 0.5
(*R*,*S*)**-5**	0.65 ± 0.03	65 ± 2.5	18.7 ± 0.6	76 ± 1.5
(*R*)**-5**	0.75 ± 0.05	48 ± 2.7	25.1 ± 1.8	82 ± 2.3
(*R,S*)**-6**	0.71 ± 0.02	40 ± 2.2	8.8 ± 0.7	40 ± 2.7
(*R,S*)**-7**	0.51 ± 0.05	62 ± 2.3	7.8 ± 0.8	35 ± 2.5
RGZ	0.24 ± 0.07	100 ± 0.2	9.8 ± 0.4	57 ± 3.1

We then evaluated the antiproliferative potential of the compounds by carrying out viability assays in HT-29, a CRC-derived cell line selected as it expresses substantial amounts of PPARγ1 with respect to the α and β/δ subtypes. The various compounds were tested in a concentration range (1 to 25 µM) centered around the IC_50_ values as also determined in preliminary experiments (Supplementary Fig. S1). Interestingly, all molecules exhibited an antiproliferative potential that ranged between 31–82%, while it was about 60% for RGZ (Table 2). On the basis of these results, we decided to analyze in further details (*R*,*S*)**-3**, (*S*)-**3** and (*R*,*S*)**-7** as they combine a limited transactivation (efficacy ranging between 55 and 65%) with the highest antiproliferative potential (31–47% of residual vitality), in comparison with the potency of all others compounds. Indeed, the EC_50_ and IC_50_ values for (*R*,*S*)-**3**, (*S*)-**3** and (*R*,*S*)-**7** are largely lower than those of the remaining compounds listed in Table 2.

### Compounds (*R*,*S*)-**3**, (*S*)-**3** and (*R*,*S*)-**7** affect cell cycle progression

**Figure 1:**
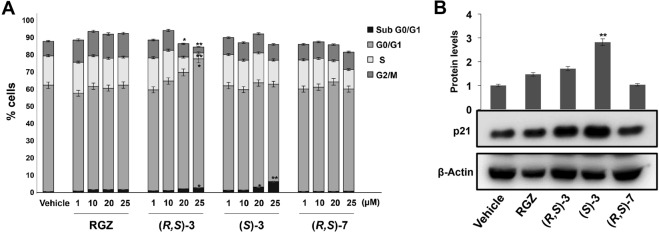
Cell cycle analysis of HT-29 cells treated with increasing amounts of RGZ, (*R,S*)**-3**, (*S*)**-3** or (*R,S*)-**7** and p21^waf1/cip1^ expression evaluation by Western blotting. (**A**) Flow cytometric assay carried out on HT-29 cells treated or not with the indicated compounds at different concentrations ranging from 1 to 25 µM for 48 hs. HT-29 cells exposed or not to these compounds were harvested, permeabilized and stained with propidium iodide and analyzed by FACS. Data are means ± SD of two independent experiments. *p ≤ 0.05, **p ≤ 0.01 compared to the control. (**B**) Proliferating HT-29 cells were treated or not for 48 hs with 10 µM of the indicated compounds. Protein extracts were prepared and assessed for p21^waf1/cip1^ expression in Western blotting analysis. β-Actin was used for protein load normalization. The bar graphs are the mean ± SD of three independent experiments. **p ≤ 0.01 compared to the control.

We then investigated the cell cycle changes underlying the growth inhibition caused by (*R*,*S*)**-3**, (*S*)-**3** and (*R*,*S*)**-7** treatment. To this goal, HT-29 cells were cultured in proliferating medium for 24 hs and exposed for additional 48 hs to a medium containing different concentrations of the three compounds. Flow cytometry analysis revealed that exposure to (*R*,*S*)-**3**, from 1 to 25 μM, caused a dosage-dependent increase of the G0/G1 cell population and a concomitant decrease of the S- and G2/M phase cells with respect to RGZ treated cells (Fig. 1A). (*S*)-**3** induced a significant increase of cells in the sub G0/G1 phase, while no significant variations were observed in the G0/G1, S and G2/M cell populations in cells treated with (*R*,*S*)-**7**.

**Figure 2:**
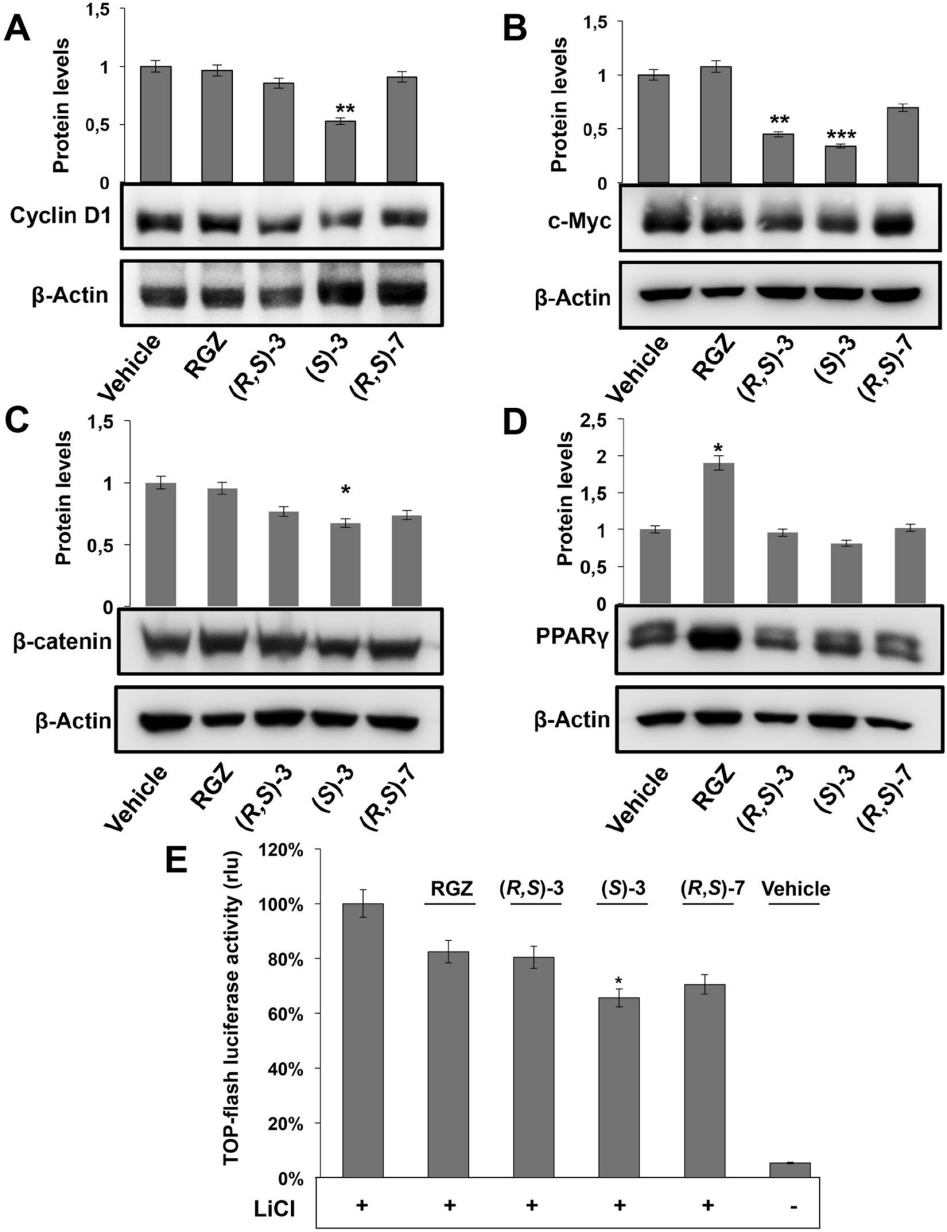
Effects of RGZ, (*R*,*S*)-**3**, (*S*)-**3** and (*R*,*S*)-**7** on the expression of different protein markers. Proliferating HT-29 cells were treated for 48 hs with 10 µM of the indicated compounds. Specific antibodies against cyclin D1 (**A**), c-Myc (**B**), β-catenin (**C**) and PPARγ (**D**) respectively, were used in Western blotting analysis. An anti-β-actin antibody was used as a control for protein loading. The graphs of (**A**) and (**B**) represent the means ± SD of three independent experiments. **p ≤ 0.01, ***p ≤ 0.005 compared to the control. The bar graphs of (**C**,**D**) are the means ± SD of three independent experiments. *p ≤ 0.05 compared to the control. **(E)** Top-FLASH luciferase assay performed in HEK-293 cells transiently transfected with the Top-FLASH reporter plasmid and exposed to 20 mM LiCl alone or in combination with 1 µM RGZ, (*R*,*S*)-**3**, (*S*)-**3**, (*R,S*)-**7** or the vehicle alone (DMSO) for 24 hs is shown. Luciferase activity is reported as fold induction after normalization to β-galactosidase activity used as control of transfection efficiency. The graph represents the mean ± SD of two independent experiments performed in triplicate. *p ≤ 0.05 compared to LiCl exposure.

To better characterize the growth inhibition elicited by (*R*,*S*)**-3**, (*S*)-**3** and (*R*,*S*)**-7**, we examined changes in the expression of proteins involved in cell cycle control (Figs 1B and 2A). To this goal, protein extracts from proliferating HT-29 cells, treated for 48 hs with the vehicle alone (DMSO), or 10 μM RGZ, (*R*,*S*)**-3**, (*S*)-**3** or (*R*,*S*)**-7**, respectively, were analyzed by Western blotting for p21^waf1/cip1^ expression. (*S*)-**3** induced a robust increase, significantly higher than the slight one obtained by RGZ and (*R*,*S*)**-3**, while (*R*,*S*)**-7** had no effect (Fig. 1B). Consistently, (*S*)-**3** caused a cyclin D1 reduction more evident than that produced by RGZ and (*R*,*S*)**-3**, while (*R*,*S*)**-7** had no effects (Fig. 2A). To verify whether these results were dependent on active transcription of these genes, we performed qRT-PCR analysis on total RNA extracted from treated and untreated cells (Supplementary Fig. S2). The results paralleled those of the corresponding proteins and all together correlated with the flow cytometric data obtained in the same experimental conditions as reported in Fig. 1.

### (*R,S*)-**3** and (*S*)-**3** interfere with the β-catenin/TCF pathway and further arrest cell proliferation

Cyclin D1 downregulation suggests that the investigated compounds may affect the β-catenin/TCF pathway. Indeed, cyclin D1 and c-Myc are target genes^38,39^ and, for these reasons, we evaluated c-Myc levels in untreated and treated HT-29 cells. (*R*,*S*)**-3**, (*S*)-**3** caused reduction of the protein, that was modest with (*R*,*S*)**-7** and null with RGZ (Fig. 2B), suggesting that only the first two compounds can counteract the β-catenin/TCF pathway. Along this reasoning, we investigated β-catenin levels in the same experimental conditions. Of note, HT-29 cells have elevated levels of β-catenin because the corresponding gene, *CTNNB1*, is mutated and the pathway is constitutively activated; this implies high and steady levels of the protein^40^. Compounds (*S*)-**3** and (*R*,*S*)**-3** caused reduction of β-catenin levels that were only slight with (*R*,*S*)**-7** and null with RGZ (Fig. 2C). qRT-PCR analysis of the corresponding mRNA levels confirmed the results, in line with those on cyclin D1 and c-Myc reported above (Supplementary Fig. S2).

To definitely confirm that (*R*,*S*)**-3** and (*S*)-**3** negatively affect c-Myc and cyclin D1 protein levels through down-regulation of their own gene transcription, we performed the TOP-flash reporter luciferase assay in LiCl-induced HEK-293 cells. Lithium chloride is known to inhibit glycogen synthase kinase-3β activity resulting in increase of both cytosolic and nuclear β-catenin levels and, hence, in enhanced β-catenin/TCF complex-mediated transcription^41^. The TOP-flash reporter plasmid contains three copies of the T-cell factor binding site (a target of the Wnt/β-catenin pathway) upstream to a promoter that drives luciferase gene transcription. Assessment of luciferase activity in the extracts of transfected cells is a means to evaluate the activity of the pathway. In untreated cells, luciferase activity was very low, as the pathway is in an “off” mode, while in LiCl treated cells, it was high, as the pathway switched to an “on” mode (Fig. 2E). Co-treatment of LiCl with (*S*)-**3** and (*R,S*)-**7** reduced the luciferase activity to about 60% and 70%, respectively, whereas (*R*,*S*)**-3** and RGZ only to 80%. These results demonstrate that (*S*)-**3**, (*R,S*)**-3** and (*R,S*)-**7** contribute to inhibit cell proliferation through a transcriptional block of the β-catenin/TCF target genes as documented by the reduced c-Myc, cyclin D1 and β-catenin protein levels. We previously showed that PPARγ plays a central role in the export of β-catenin to the cytoplasm and subsequent degradation^42^; this may explain the result reported here (Fig. 2C,D).

### (*S*)-**3** stimulates apoptosis, while (*R*,*S*)-**3** promotes cell cycle arrest of HT-29 cells

**Figure 3:**
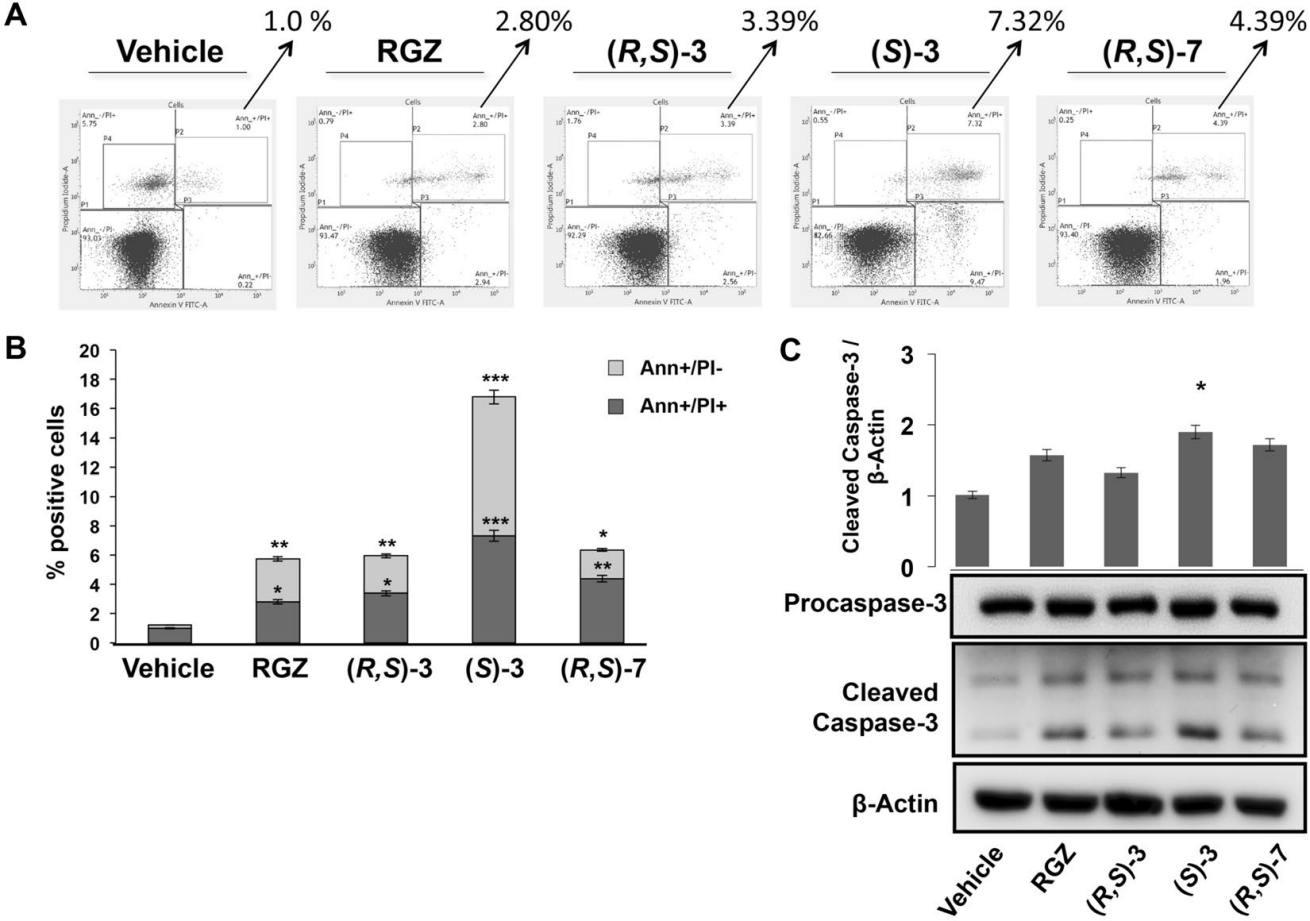
Effects of RGZ, (*R*,*S*)-**3**, (*S*)-**3** and (*R*,*S*)-**7** on HT-29 cell apoptosis and caspase-3 activation analysis. (**A**) HT-29 cells were treated for 48 hs with 10 µM of RGZ, (*R*,*S*)-**3**, (*S*)-**3** and (*R,S*)-**7**, respectively, stained by Annexin V-propidium iodide and analyzed by flow cytometry. (**B**) Early (Ann+/PI−) and late (Ann+/PI+) apoptotic cell populations were evaluated and reported in the graphic representation. The bar graphs are the means ± SD of three independent experiments. *p ≤ 0.05, **p ≤ 0.01, ***p ≤ 0.005 compared to the control. (**C**) Total protein extracts from proliferating HT-29 cells, treated or not with 10 µM RGZ, (*R*,*S*)-**3**, (*S*)-**3** and (*R,S*)-**7**, respectively, for 48 hs were analyzed by Western blotting with an anti-caspase 3 antibody. An anti-β-actin antibody was used as a control for protein loading. Significance is indicated as *p ≤ 0.05 compared to only vehicle.

To evaluate whether the cell cycle arrest induced by our compounds was accompanied by induction of cell death, we treated HT-29 cells with 10 μM RGZ, (*R*,*S*)**-3**, (*S*)-**3** and (*R*,*S*)**-7** for 48 hs and then stained with Annexin V-FITC and propidium iodide followed by flow cytometry analysis (Fig. 3A,B). All compounds stimulated apoptosis; the percentage of Annexin^+^/propidium^+^ cells was higher in (*S*)-**3** than RGZ, (*R*,*S*)**-3** and (*R,S*)-**7** treated cells. Accordingly, we surveyed the levels of caspase 3, one of the effectors of the process, and found a significant reduction of the precursor and a concomitant increase of the cleaved form with (*S*)-**3** exerting the strongest effect (Fig. 3C). These results are in accordance with flow cytometry that showed a time- and dose-dependent appearance of a sub G0/G1 peak, a typical feature of apoptosis (Fig. 1A).

### The antiproliferative activity of (*R*,*S*)-**3**, (*S*)-**3** and (*R,S*)-**7** is dependent upon PPARγ activation

**Figure 4:**
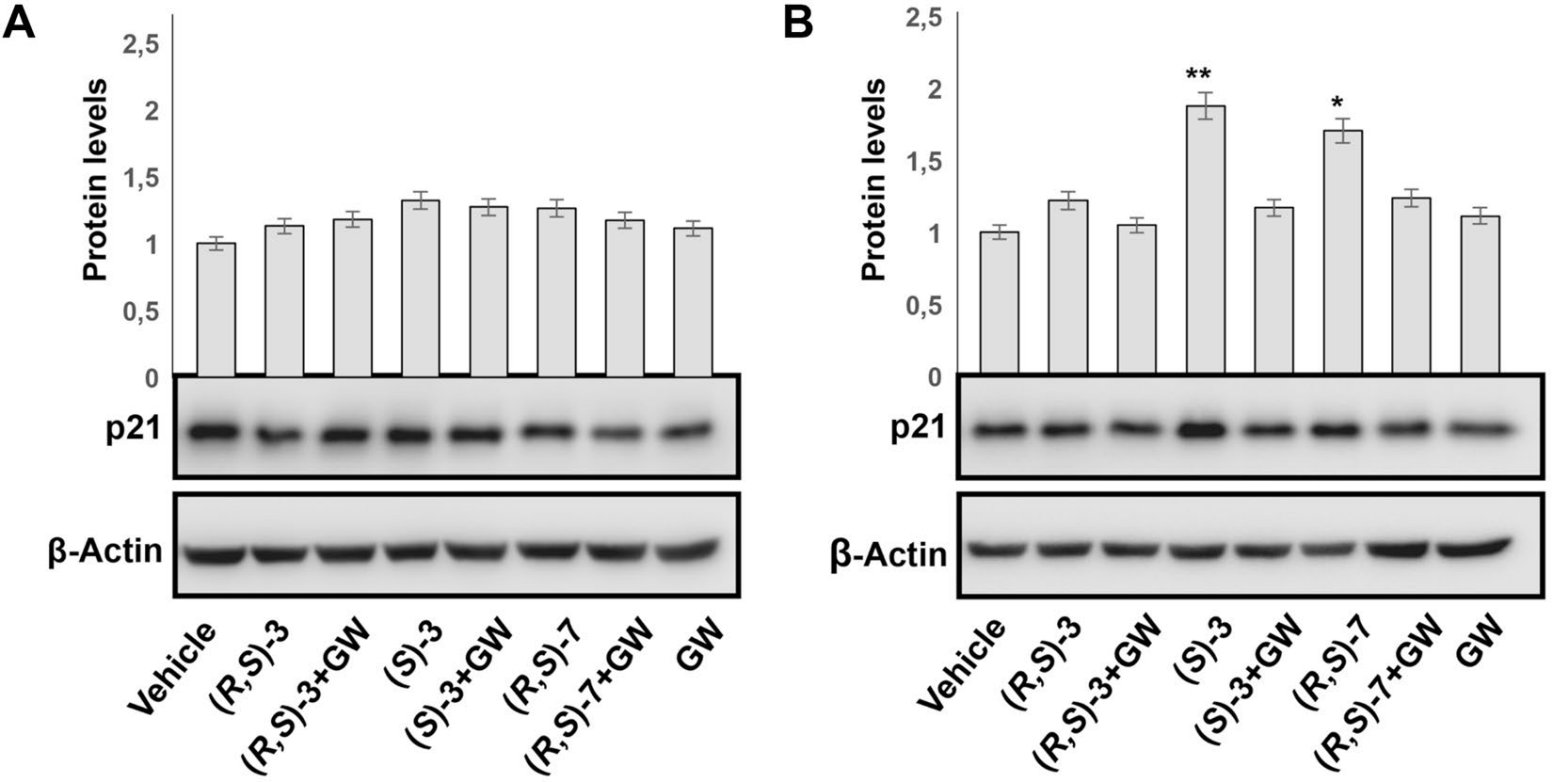
PPARγ-dependent antiproliferative effect in CRC-derived RKO cells. Western blotting analysis of p21^waf1/cip1^ expression in total protein extracts from CRC-derived RKO cells (**A**) and its derived clone overexpressing an ectopic PPARγ1 (**B**) using an anti-p21^waf1/cip1^ antibody. An anti-β-actin antibody was used as a control for protein loading. The bar graphs represent the mean ± SD of PPARγ/β-actin of at least 3 independent experiments. *p ≤ 0.05, **p ≤ 0.01 compared to the control.

To finally demonstrate that the cell proliferation arrest detected with the indicated compounds occurs in a PPARγ-dependent manner, we analyzed protein extracts from RKO cells stably transfected with an expression vector for *PPARG1* and treated with 10 μM (*R*,*S*)**-3**, (*S*)-**3** and (*R,S*)-**7** for 48 hs, in the presence or absence of GW9662, an irreversible PPARγ antagonist. We chose the CRC-derived RKO cells as they do not express the endogenous *PPARG*; the cell clone used expresses, instead, a fixed amount of the exogenous receptor to which the results obtained can be referred. RKO cells were taken as control for assessing possible effects due to other PPAR receptors expressed at low levels in this cell or to PPARγ-independent effects elicited by GW9662, as reported^36^. As illustrated in Fig. 4A,B, the various compounds induced accumulation of p21^waf1/cip1^ while GW9662 treatment reverted the effect. The same analysis showed no p21^waf1/cip1^ variations in RKO cells, suggesting that the effects produced by (*R*,*S*)-**3**, (*S*)-**3** and (*R,S*)-**7** occur through a PPARγ dependent-mechanism; we also rule out that they can be mediated either by the other PPAR subtypes or by GW9662.

### Docking studies suggest the binding pose of compound (*S*)-**3** into PPARγ LBD

The experiments reported here suggest that, out of the entire series, (*S*)-**3** is the most active compound. In order to understand the structural basis for its recognition by PPARγ, we carried out docking studies using the X-ray crystal structure of PPARγ in complex with the partial agonist (2*S*)-2-(4-chlorophenoxy)-3-phenylpropanoic acid (PDB code: 3CDP)^43^. This structure was chosen because of the good resolution (2.8 Å) and the similarity of the co-crystallized ligand with the compound series. Docking was performed using Glide module, which is part of the Maestro software suite^44,45^.

The docking protocol was standardized before performing any further study. To this aim, the co-crystallized ligand was prepared with LigPrep module and re-docked. On comparing the conformation of the co-crystallized ligand with the docked poses, it was observed that SP (standard precision) mode reproduced the bioactive conformation of the bound ligand (with RMSD less than 2 Å). Thus, further molecular docking studies were performed at SP level.

The PPARγ LBD is an approximately Y-shaped hydrophobic cavity formed through the contribution of H3, H5, H6, H7, H11, H12 and the β-sheet. Several full agonists, such as RGZ, occupy a region of this cavity extending from the β-sheet to the activation AF-2 domain, whereas several partial agonists such as nTZDpa^46^ and intermediate agonists such as BVT.13^47^ occupy only the region proximal to the β-sheet and H3.

**Figure 5:**
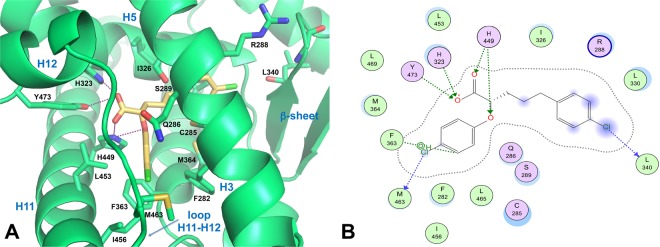
Docking of compound (*S*)-**3** into the PPARγ binding pocket. (**A**) Binding mode of representative compound (*S*)-**3** (a partial agonist, yellow sticks) into the PPARγ LBD represented as a limegreen ribbon model. Only amino acids located within 4 Å of the bound ligand are displayed and labeled. H-bonds discussed in the text are depicted as dashed deep-purple lines. (**B**) 2D ligand-interaction diagram of (*S*)-**3** into the PPARγ LBD generated by the MOE software package. Green spheres = “greasy” residues; spheres with red outline = acidic residues; spheres with blue outline = basic residues; spheres with black outline = polar residues; blue background spheres = receptor exposure to solvent; blue spheres on ligand atoms = ligand exposure to solvent. Green dotted lines = side chain donors/acceptors; blue dotted lines = halogen contact; grey dotted line = proximity contour.

Docking of (*S*)-**3** into the PPARγ LBD revealed that the binding mode of this compound was similar to that previously reported for other α-aryloxy-β-phenylpropanoic acids, in that it occupies not only the cavity proximal to the AF-2 surface but also, partially, that proximal to the β-sheet and H3 region^43,48^ (Fig. 5A,B). The carboxylate moiety of the ligand establishes the canonical intermolecular H-bonding network with the residues that are generally involved in the binding of carboxylate-containing ligands. In particular, one of the carboxylate oxygens forms a H-bond with the H449 N^ε2^ atom (2.9 Å), which, in turn, engages the ligand ether oxygen in a further H-bond (3.2 Å). The other carboxylate oxygen forms a bifurcated H-bond with the H323 N^ε2^ (2.7 Å) and Y473 OH atoms (3.6 Å) located on the AF-2 domain. However, this latter interaction is weak because of a large donor-acceptor distance (3.6 Å).

The *p*-Cl-phenoxy ring occupies the diphenyl pocket, a region of the LBD proximal to the activation function-2 (AF-2) that includes H11, H3 and loop 11/12 and can accommodate ligands with long and straight substituents^49,50^. Inside the cavity, the *p*-Cl-phenoxy group makes van der Waals contacts with L453 and I456 of H11, F363 of H7, and F282 and Q286 of H3. Notably, the Cl atom is in weak halogen bonding distance to the sulphur atom of M463 side chain on the loop 11/12 (Cl···S distance = 3.9 Å, C−Cl···S angle = 149°), contributing in this way to stabilize the loop 11/12 positioning. It is well known that differences in the hydrophobic packing of this loop may contribute to diverse H12 dynamics^51^.

The *p*-Cl-phenylpropyl moiety of the ligand lies between H3, H7 and H5, allowing for their stabilization through hydrophobic contacts. Specifically, this moiety makes hydrophobic contacts with the side chains of R288 (H3), C285 (H3), M364 (H7), I326 (H5) and L330 (H5). In addition, the Cl atom makes a weak halogen bond with the carbonyl oxygen of L340 main chain on the β-sheet (Cl···O = 4 Å, C−Cl···O angle = 151°), allowing for better packing within the ligand-binding pocket through van der Waals interactions.

Superposition of the docking pose of (*S*)-**3** over the co-crystallized parent partial agonist (2*S*)-2-(4-chlorophenoxy)-3-phenylpropanoic acid (PDB ID: 3CDP)^43^ showed that both carboxylic groups and *p*-Cl-phenoxy ring moieties nicely overlap (Supplementary Fig. S3). Hence, (*S*)-**3** shares the same pattern of interactions as (2*S*)-2-(4-chlorophenoxy)-3-phenylpropanoic acid in the branch I of PPARγ (the H12 subpocket) and is believed to trigger the same receptor structural dynamics^48,52,53^.

## Discussion

In this study we tested the ability of some chiral phenoxyacetic acid analogues (compounds **1**–**7**) to effectively act as PPARγ ligands. These compounds belong to a previously reported series of PPARα/γ dual agonists. Specifically, compounds **1**–**7** acted as PPARα full agonists and PPARγ partial agonists in transactivation assays^37^. Here, we confirmed this last result with a different transactivation assay based on the transfection of a full-length PPARγ1 cDNA and a luciferase reporter construct under the control of specific PPREs. In these experimental conditions, the entire group of compounds exhibited a transactivation potency, in terms of EC_50_, similar to a full agonist as RGZ, while the efficacy was definitely lower, ranging between 40 and 65% of RGZ, in line with other known partial agonists. Interestingly, we also showed that compounds **1**–**7** are endowed with antiproliferative activity in CRC cells, chosen as they express PPARγ more than PPARα and PPARβ/δ. The entire series displayed a variable efficacy ranging between 31 and 82% of residual vitality, i.e. remaining surviving cells, with respect to the 57% produced by the full agonist RGZ. Compounds (*R,S*)-**3**, (*S*)-**3** and (*R,S*)-**7** combined a transactivation efficacy lower than a full agonist (65%, 55% and 62%, respectively, with an EC_50_ 0.35, 0.40 and 0.51 µM, respectively, slightly higher than RGZ 0.24 µM) with a remarkable antiproliferative activity (47%, 31% and 35% of residual viability, respectively, with IC50 values of 7.3, 4.8 and 7.8 µM compared to 9.8 µM obtained by RGZ). For these reasons, they were selected and further surveyed. The three indicated molecules caused, indeed, a G1-phase arrest with increase of the specific cell cycle regulator p21^waf1/cip1^ and simultaneous decrease of cyclin D1. The cell cycle block was accompanied by induction of apoptosis as documented by flow cytometry and caspase activation (Figs 1–3). We also demonstrated that the effects are mediated through a PPARγ-dependent mechanism (Fig. 4) and ruled out that they can be referred to PPARα or PPARβ/δ modulation or to a PPARγ-independent effect, at least in the cell system used. This lack of PPARα or β/δ activity suggests that these compounds might transactivate the diverse PPAR subtypes in a cell-specific manner; alternatively, the result might be due to different sets of coactivators or corepressors present in the cell context analyzed.

Another intriguing feature of the three investigated compounds, especially (*S*)-**3**, is that they interfered with the Wnt/β-catenin/TCF pathway (Fig. 2C–E) that plays a crucial pathogenetic role both in familial and sporadic CRCs. By different experimental approaches, we provided evidence that (*S*)-**3** reduces β-catenin and, thus, its ability to stimulate transcription of cyclin D1 and c-Myc, target genes involved in cell cycle control and growth (Fig. 2). The characteristics of the compounds analyzed here are in line with the ongoing intense investigations aimed to use them in CRC therapy.

The interactions of a specific ligand with the aminoacids lining the LBD of NRs lead to conformational changes involving the AF-2 helix (i.e., helix H12), which switches the receptor from the “off” to “on” status^54^. This structural plasticity is usually a consequence of the binding to full agonists; alternative dynamic activation models of the LBD have been proposed, among which very attractive are those due to the binding of partial agonists. These ligands, indeed, partially stabilize the AF-2 surface that includes H3, H3-4 loop, H11 and H12 and stimulate a limited transcriptional activity^55^. Thus, the degree and the type of such interactions cause stabilization or destabilization of extended surfaces of the LBD exposed to the binding with coactivators or corepressors necessary for the assembly and functionality of the transcriptional machinery^56^. In keeping with this reasoning, investigations on natural and synthetic PPARγ ligands are underway to identify molecules with different binding modes that can recruit different subsets of coactivators/corepressors activating distinctive downstream pathways^57^. The differential recruitment might also explain the absence or reduction of the adverse side effects associated with full agonists.

We recently characterized cladosporols, a group of natural PPARγ ligands, which promote a strong antiproliferative and proapoptotic activity in CRC cells^58^. Specifically, cladosporol B, an oxidate form of cladosporol A, displays a strong antiproliferative activity, low affinity for the PPARγ LBD and reduced PPRE-mediated transactivation potential as compared to cladosporol A. The structures of the PPARγ-LBD in complex with both cladosporols A or B provided a molecular rationalization of their behavior as full or partial agonist, respectively. In addition, cladosporol B showed stronger antiproliferative and proapoptotic activity in CRC cells compared to cladosporol A allowing to hypothesize that a correlation could exist between these effects and the different binding to PPARγ LBD^58^. Also the compounds investigated here display reduced transactivation capacity consistent with being partial agonists and, as cladosporol B, are endowed with a stronger antiproliferative activity compared to the full agonist RGZ (Figs 1–4). Ligand docking experiments revealed that the binding mode and the receptor interaction pattern of the most potent compound (*S*)-**3** resembles that of the parent partial agonist (2*S*)-2-(4-chlorophenoxy)-3-phenylpropanoic acid co-crystallized with PPARγ (PDB ID: 3CDP)^43^ (Supporting Information, Fig. S3). In details, (*S*)-**3** interacts with the three residues H323, H449, and Y473 (Fig. 5), usually recognized as pivotal in the binding of carboxylate-containing ligands and involved in receptor activation. However, the distance between the acidic moiety of (*S*)-**3** and Y473 in H12 is quite long, accounting for a milder stabilization of H12. Furthermore, the *p*-Cl-phenoxy ring of (*S*)-**3** is deeply inserted into the diphenyl pocket, between H11, H3 and the loop 11/12, thereby forming several favorable hydrophobic interactions. Thus, (*S*)-**3** stabilizes preferentially H3 through closer hydrophobic contacts with residues of this helix, losing the characteristics of full agonist and acquiring those of partial agonist. This relationship is in agreement with our previous findings regarding the crystal complexes of PPARγ and two enantiomeric ureidofibrate-like derivatives. Even in that case, while the full agonism of one enantiomer could be related to stronger interactions with H11, H12, and the loop 11/12, the partial agonism of the other enantiomer could be ascribed to closer contacts with the residue Q286 of H3^59^. Interestingly, both enantiomeric ureidofibrate-like derivatives, similarly to the phenoxyacetic acid analogues reported here, potently inhibited cellular proliferation in CRC cell lines and effectively induced apoptosis in cancer cells. Therefore, it can be speculated that compounds with distinct molecular structures, acting as PPARγ partial agonists and inducing growth inhibition and apoptosis, might share a common binding pattern. Further studies are needed to firmly establish a possible correlation between this mode of binding and the activation of the same selected pathways. It would be deemed to explore whether the differences induced in the 3D structure of PPARγ are linked to recruitment of diverse transcriptional cofactors that, in turn, would produce differential results.

In conclusion, the investigation of a series of compounds showing different biological properties and a distinct mode of binding to the PPARγ-LBD will certainly improve our understanding of the pharmacological profile that distinguishes full from partial PPARγ agonists, allowing the identification of a more favorable ratio between beneficial and adverse effects.

## Materials and Methods

### Cell culture, antibodies and reagents

The human colon adenocarcinoma derived cells HT-29, RKO and human kidney HEK293 were obtained from the American Type Culture Collection (Rockville, MD, USA). HT-29 cells bear different genetic abnormalities typical of human CRC, such as a mutated Tp53 (Arg 273 His) and a wild-type RAS allele. Antibodies against p21waf1/cip1, Cyclin D1, PPARγ, β-actin and anti-mouse and anti-rabbit IgG peroxidase-linked secondary antibodies were purchased from Santa Cruz Biotechnology (Santa Cruz, CA, USA). Anti-caspase-3 and anti-c-Myc were obtained from Cell Signaling Technology (Danvers, MA, USA). Antibodies against E-cadherin and β-catenin were from BD transduction (San Jose, CA, USA). ECL and ECL Plus Western blotting detection kit were from Bio-Rad (Hercules, CA, USA). D-MEM (Dulbecco’s Modified Eagle’s Medium), D-luciferin sodium salt, RGZ and GW9662 were from Sigma Aldrich (St.Louis, MO, USA). Foetal bovine serum (FBS), penicillin-streptomycin, L-glutamine, trypsin-EDTA and OptiMEM I were obtained from Gibco (Carlsbad, CA, USA). Lipofectamine 2000 were from Thermo Fisher (Waltham, MA, USA).

### Cell culture and RGZ, (*R*,*S*)-**3**, (*S*)-**3** and (*R,S*)-**7** treatments

HT-29, RKO and HEK293 cell lines were grown as a monolayer in D-MEM containing 10% FBS, 1% penicillin-streptomycin and 1% L-glutamine. The cells were cultured in 100 mm plates, at 70–80% confluence, in a 5% CO2 humidified atmosphere, at 37 °C. (*R*,*S*)**-3**, (*S*)-**3** and (*R,S*)-**7** were dissolved in DMSO and mixed with fresh medium to achieve the final concentration. In all treatments, the DMSO final concentration in the medium was less than 0.1%. In any experiments cells were treated with different concentrations of (*R*,*S*)**-3**, (*S*)-**3**, (*R,S*)-**7**, RGZ and 0,1% DMSO were used as control.

### Cell viability

To analyze the growth rate of HT-29 after exposure to RGZ, (*R*,*S*)**-3**, (*S*)-**3**, (*R,S*)-**7**, cells were plated in 24-well plates at density of 50000 cells/cm^2^. After treatment, the cells were washed with PBS, trypsinized and collected in culture medium. Cells were counted by means of a Burker’s hemocytometer and by automated cell counter (Roche Applied, Penzberg, Germany)^60^.

### Flow cytometry analysis

HT-29 cells were plated at similar confluency and synchronized by a 48 h serum deprivation in 0.1% FBS. An aliquot of the cells was treated with 1–10–20 and 25 μM of (*R*,*S*)**-3**, (*S*)-**3**, (*R,S*)-**7** and RGZ for 48 h in the presence of DMEM containing 10% FBS. At same time, another aliquot of cells was allowed to grow again in the complete medium containing 0,1% DMSO for the same times and was used as control. After treatment, DNA cell content was evaluated by FACS analysis as previously described^60^. Evaluation of apoptotic cells was performed by staining cell treated or not with 10 μM of the indicated compounds for 48 hs with Annexin5-V-Fitc and propidium iodide. All flow cytometry results were analyzed by FACSuite Software.

### Western blotting analysis

Total extracts from treated and untreated cells were obtained by lysis in Ripa buffer (150 mM NaCl, 50 mM Tris-HCl, pH 7.6, 10 mM EDTA, 1% NP-40) containing also a protease inhibitors cocktail and then centrifugated at 17,000 RCF for 10 min, at 4 °C. Total proteins in the supernatant were quantified and 80 μg of each sample were loaded on SDS-PAGE. Western blotting assays were carried out as previously reported^60^.

### Plasmids and transient transfection experiments

HEK293 cells that stably express an exogenous Flag-tagged PPARγ1 from a transfected PCDNA-3 vector carrying a complete PPARγ cDNA were used for transfection experiments. PPRE-Luc plasmid contains a luciferase reporter gene whose transcription is driven by the herpes simplex thymidine kinase (TK) promoter including three copies of the PPRE designed on the sequence of Acyl-CoA oxidase gene. In these transient transfection assays, we used, as internal control of efficiency, the RSV-βGal plasmid, expressing β-galactosidase gene under the control of the strong Rous Sarcoma Virus (RSV) promoter. The day before transfection, HEK293 stably expressing FLAG-PPARγ1 were plated in 12-well plates to reach 70% confluence. After 24 hs, growth medium was removed and substitute with OPTI-MEM, in absence of serum and antibiotics, and cells were transfected with the luciferase reporter gene (PPRE-Luc) using lipofectamine 2000 reagent as described^60^. About 10–12 hs after transfection, cells were washed and treated with different concentrations of (*R*,*S*)**-3**, (*S*)-**3**, (*R,S*)-**7** and RGZ. Transfection assays were performed in triplicate and the resulting transcriptional activities measured by luciferase assay. The values were normalized by β-galactosidase assay and the average value for each triplicate was calculated^60^.

### Top-FLASH reporter assay

The Top-FLASH reporter plasmid containing three copies of TCF binding sites was transiently transfected into HEK293 cells with Lipofectamine 2000 (Invitrogen, Carlsbad, CA, USA) in 24-well plates. To activate the Wnt/β-catenin pathway, cells were treated with 20 mM LiCl alone or in combination with 1 µM (*R*,*S*)**-3**, (*S*)-**3**, (*R,S*)-**7** and RGZ for 24 hs. Luciferase activity was measured after 24 h of compounds treatment and normalized to β-galactosidase. All experiments were performed two times in triplicate.

### Real-Time Quantitative PCR (RT-qPCR) Assays

RNA was isolated from treated and untreated HT-29 cells using TRIZOL reagent according to the manufacturer’s instructions. After the control of purity, integrity, and concentration of total RNA by gel electrophoresis and UV spectroscopy, cDNAs were obtained using SuperScriptTM II reverse transcriptase (Invitrogen, Carlsbad, CA) from 2 mg of total RNA as already described^60^.

qRT-PCR performed on QuantStudio5 (Applied Biosystems), using PowerUp SYBR Green Master Mix (Invitrogen). Expression of each gene was standardized using 18S RNA as reference, and relative levels were quantified calculating 2^ΔΔ*C*^_T_, where ΔΔ*C*_T_ is the difference in *C*_T_ (cycle number at which the amount of amplified target reaches a fixed threshold). The results are the mean of at least two independent experiments. Specific primers were used to analyze the transcription of the different genes, as p21waf1/cip1: 5′-GACACCACTGGAGGGTGACT-3′ and 5′-AGGTCCACATGGTCTTCC-3′; β-catenin gene: 5′-CCAGCGTGGACAATGGCTAC-3′ and 5′-TGAGCTCGAGAGTCATTGCATAC-3′;c-Myc 5′-CACCAGCAGCGACTCTGA3′ and 5′-GATCCAGACTCTGACCTTTTGC3′; cyclinD1:5′-CCGTCCATGCGGAAGATC-3′ and 5′-ATGGCCAGCGGGAAGAC3′; PPARγ: 5′-CGTGGCCGCAGATTTGAA-3′ and 5′-CTTCCATTACGGAGAGATCCAC3′; 18S: 5′-GGGAGCCTGAGAAACGGC-3′ and 5′-GGGTCGGGAGTGGGTAATTT-3′^60^.

### Statistical analysis of the *in vitro* assays

All experiments were performed in triplicate with three biological replicates. Data were expressed as means ± SD using the Student’s t test. P-values less than 0.05 were considered significant. Asterisks reported show significance degrees, set to *p ≤ 0.05, **p ≤ 0.01, ***p ≤ 0.005.

### Computational chemistry

Protein and ligand preparation, docking calculations and superposition were performed using Maestro 11.0 (Schrödinger, LLC, New York, NY, 2018)^61^ and UCSF-Chimera 1.8.1 (http://www.cgl.ucsf.edu/chimera) software packages^62^ running on a E4 Computer Engineering E1080 workstation provided of a Intel Core i7-930 Quad-Core processor. All the figures within the manuscript were rendered with Pymol 2.0 (Schrödinger, LLC, New York, NY, 2018).

#### Protein and ligand preparation

The starting coordinates of PPARγ in complex with the partial agonist (2*S*)-2-(4-chlorophenoxy)-3-phenylpropanoic acid (PDB: 3CDP)^43^, retrieved from Brookhaven Protein Database, were employed for the docking calculations. The protein was processed with the Protein Preparation Wizard implemented in Maestro. Hydrogen atoms were added to the protein consistent with the neutral physiologic pH. The guanidine and ammonium groups of arginine and lysine side chains were considered cationic, whereas the carboxylate groups of the aspartic and glutamic residues were considered anionic. The H-bonding network was optimized adjusting the protonation and flip states of the imidazole rings of the histidine residues together with the side chain amides of glutamine and asparagine residues. Then, the protein hydrogens atoms were energy-minimized with the Impref module, using the OPLS_2005 force field. The core structure of compound (*S*)-**3** was built by using the Molecular Builder module in Maestro. The ligand was then preprocessed with LigPrep 3.3 (Schrödinger, LLC, New York, NY, 2018) and optimized by Macromodel 10.7 (Schrödinger, LLC, New York, NY, 2018), using the MMFFs force field with the steepest descent (1000 steps) followed by truncated Newton conjugate gradient (500 steps) methods. Partial atomic charges were assigned with the OPLS-AA force field.

#### Docking simulations

Docking of (*S*)-**3** was performed with the Schrödinger Glide algorithm^44,45^. A docking grid was generated, enclosing a box centered on the native ligand with a dimension of 12 × 12 × 12 Å. A scaling factor of 0.8 was set for van der Waals radii of receptor atoms. Ligand sampling was allowed to be flexible. Default docking parameters were used, and no constraints were included. At most ten docking ligand poses were retained per run and ranked using the GlideScore function^44,45^. Binding poses were selected on the basis of the scoring, the similarity to the cocrystallized ligand binding mode and the consistency of protein-ligand interactions with the experimental data.

## Supplementary information

Supplementary Information
