# Supplementary material for: Chiral phenoxyacetic acid analogues inhibit colon cancer cell proliferation acting as PPARγ partial agonists
Source: Sci Rep. 2019 Apr 1;9:5434. doi: 10.1038/s41598-019-41765-2 (PMC6443668; doi:10.1038/s41598-019-41765-2)
Supplement: Supplementary file 1 — Supplementary Information [file 41598_2019_41765_MOESM1_ESM.docx]

**Supplementary Information**

**Chiral phenoxyacetic acid analogues** **inhibit colon cancer cell proliferation acting as PPARγ partial agonists**

Lina Sabatino,^1¶^ Pamela Ziccardi,^1¶^ Carmen Cerchia,^2¶^ Livio Muccillo^1^, Luca Piemontese,^3^ Fulvio Loiodice,^3^ Vittorio Colantuoni,^1^ Angelo Lupo^1*^, Antonio Lavecchia^2*^

^1^Dipartimento di Scienze e Tecnologie, Università del Sannio, via Port’Arsa 11, 82100 Benevento, Italy

^2^Dipartimento di Farmacia, “Drug Discovery” Laboratory, Università degli Studi di Napoli Federico II, via D. Montesano 49, 80131 Napoli, Italy

^3^Dipartimento Farmacia-Scienze del Farmaco, Università degli Studi di Bari “Aldo Moro”, via Orabona 4, 70125 Bari, Italy

*Correspondence: [lupo@unisannio.it](mailto:lupo@unisannio.it) (A.L.), [antonio.lavecchia@unina.it](mailto:antonio.lavecchia@unina.it) (A.L.)

**Supplemental Figures and Tables**

**Figure S1.** Dose-response as proliferation assay in HT-29 cells treated with RGZ, *R,S*-**3**, *S*-**3** and *R,S*-**7**.

**Figure S2.** qRT-PCR analysis of p21waf1/cip1, Cyclin D1, c-Myc, β-catenin, PPARγ mRNAs in HT-29 cells.

**Figure S3.** Cα superposition of the complexes of PPARγ with (*S*)-**3** (yellow sticks, docked pose) and (2*S*)-2-(4-chlorophenoxy)-3-phenylpropanoic acid (magenta sticks, PDB ID: 3CDP).


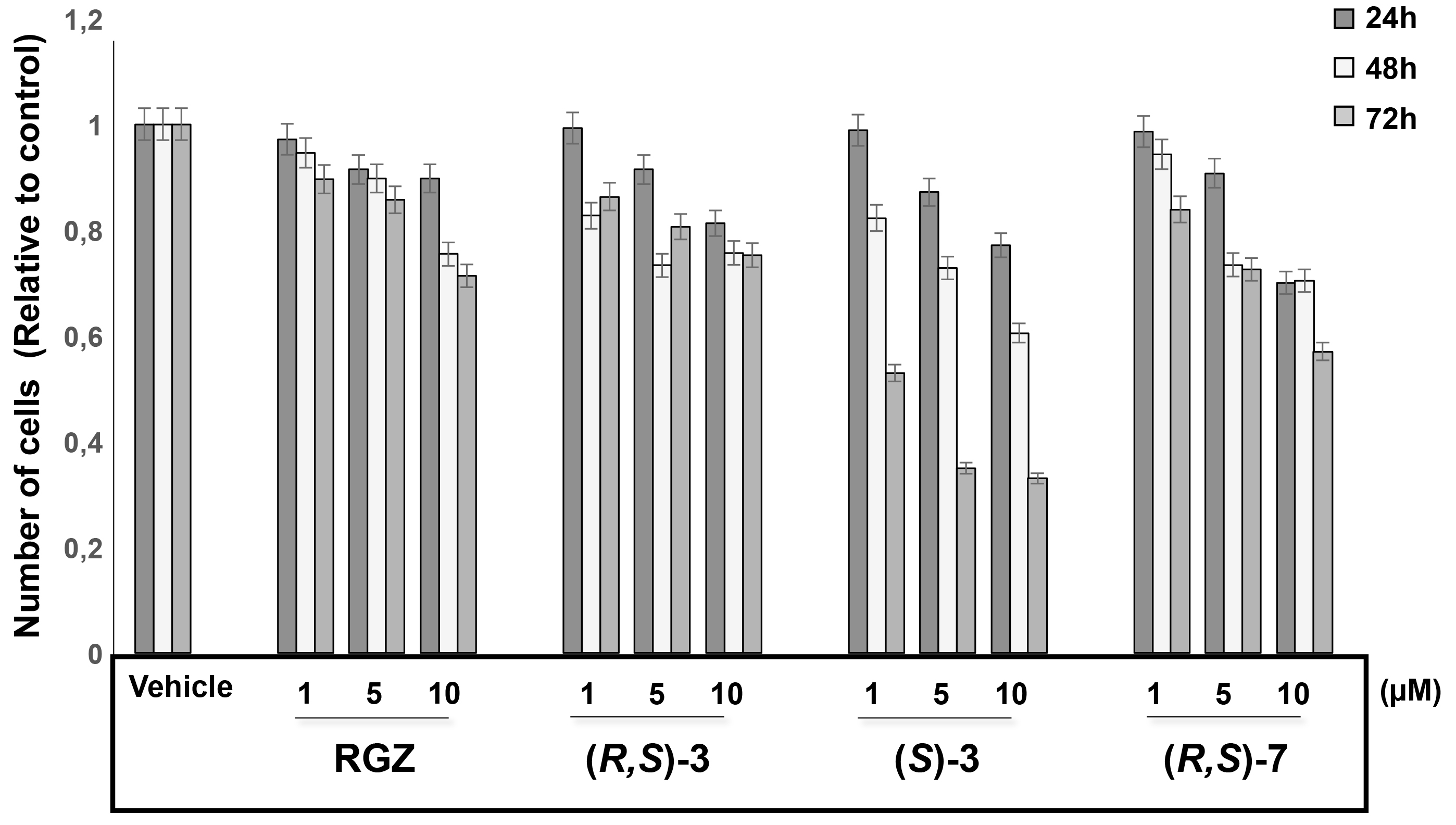


**Figure S1. Dose-response analysis of HT-29 cells treated with increasing amounts of RGZ, (*R,S*)-3, (*S*)-3 or (*R,S*)-7.** Proliferation assay carried out on HT-29 cells treated or not with the indicated compounds at different concentrations ranging from 1 to 10 µM for 48 hs. HT-29 cells exposed or not to these compounds were harvested, washed with PBS, trypsinized and collected in culture medium. Cell counting was performed by means of a Burker’s hemocytometer. Three counts for each well were made and the mean value and the standard deviation were calculated.


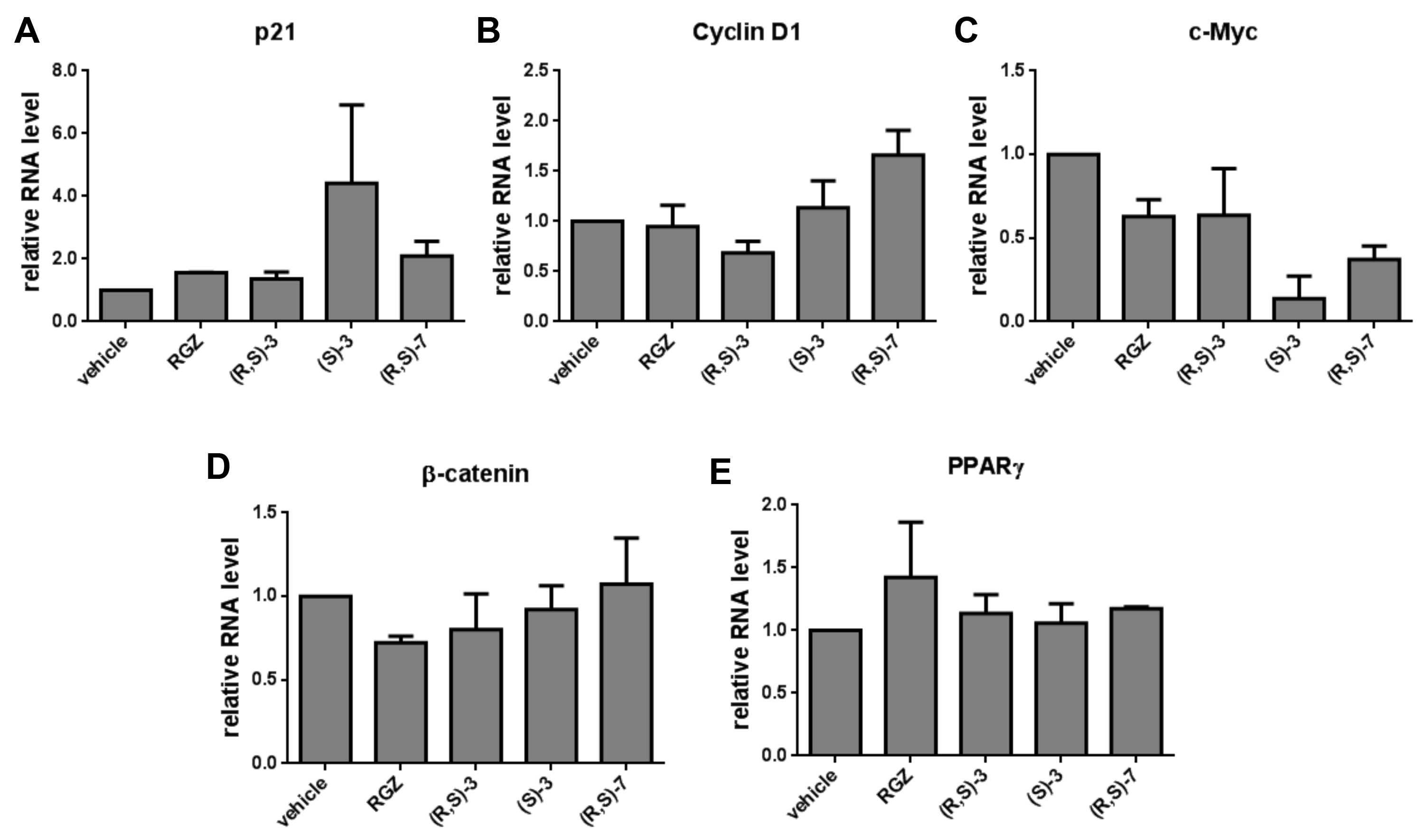


**Figure S2.** **qRT-PCR analysis** **of p21waf1/cip1 (A), Cyclin D1 (B), c-Myc (C), β-catenin (D), PPARγ (E) mRNAs in HT-29 cells.** Real-time qRT-PCR was performed using total RNA extracted from proliferating HT-29 cells treated or not with 10 µM RGZ, (*R,S*)-**3**, (*S*)-**3** or (*R,S*)-**7** for 8 h. The bar graphs represent the mean ± SD of at least two independent experiments. ***p* ≤ 0.01 compared to the control.


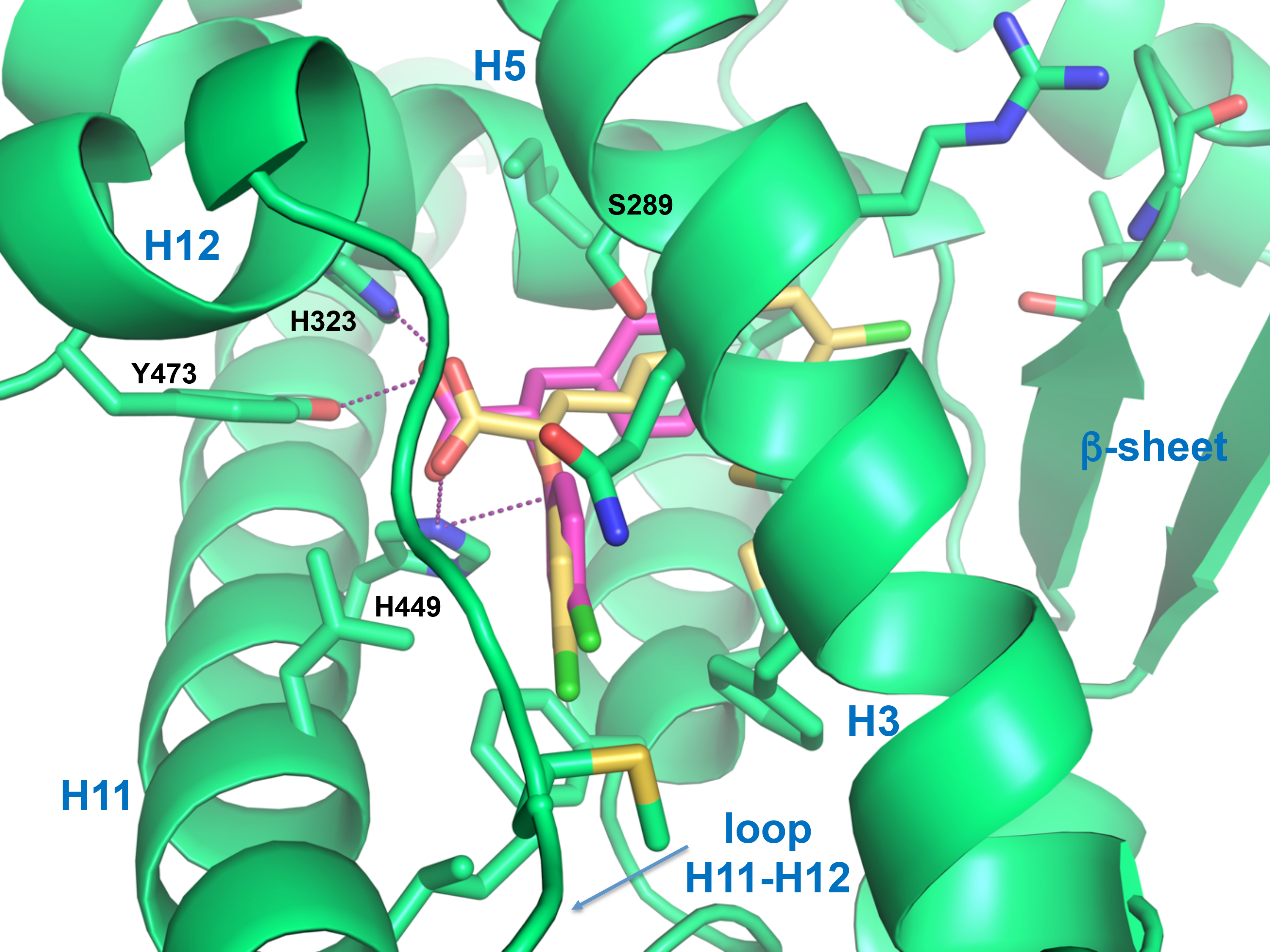


**Figure S3.** Cα superposition of the complexes of PPARγ with (*S*)-**3** (yellow sticks, docked pose) and (2*S*)-2-(4-chlorophenoxy)-3-phenylpropanoic acid (magenta sticks, PDB ID: 3CDP).
